# Incidence of, and Risk Factors and Outcomes Associated with, Acute Kidney Injury in COVID-19 at the National Kidney and Transplant Institute, Philippines

**DOI:** 10.3390/tropicalmed8080387

**Published:** 2023-07-28

**Authors:** Melchor Altillero, Romina Danguilan, Mel Hatra Arakama

**Affiliations:** National Kidney and Transplant Institute, Quezon City Metro Manila, Quezon City 1100, Philippines; maltillero@yahoo.com

**Keywords:** acute kidney injury, COVID-19, incidence, risk factors, recovery

## Abstract

(1) Background: Acute kidney injury (AKI) in COVID-19 leads to an increase in patient mortality, especially among chronic kidney disease (CKD) patients. (2) Methods: A retrospective cohort of 519 adults admitted from 1 March 2020 to 1 March 2022 were reviewed for baseline characteristics and their association with renal outcomes. Patients were divided into diagnosed CKD, undiagnosed CKD, and normal eGFR. Chronic dialysis and kidney-transplant patients were excluded. Kaplan–Meier survival analysis at 7, 14, and 30 days from admission was performed. (3) Results: The overall incidence of AKI was 45.66%; the proportions among patients with diagnosed CKD, undiagnosed CKD, and normal eGFR were 76.64%, 38.75%, and 7.59%, respectively (*p* < 0.0001). Multivariate analysis showed that being male and inotrope use were significant risk factors for AKI, while higher eGFR was protective. AKI was associated with dialysis, invasive ventilation (*p* < 0.0001), prolonged hospitalization (*p* = 0.0001), and mortality (*p* < 0.0001). Renal recovery was 64%, 59%, and 23% in stages 1, 2, and 3 AKI, respectively, until 14 days from discharge (*p* < 0.0001). Patient survival was lower in cases of AKI: 83.16%, 70.59%, and 47.5% compared to non-AKI figures of 91.27%, 87.82%, and 76.95% at 7, 14, and 30 days respectively(*p* = 0.0001). (4) Conclusion: There was a higher incidence of AKI with worsening renal function. Intensified preventive measures for AKI are crucial to prevent its devastating consequences.

## 1. Introduction

COVID-19 has been found to show a strong predilection towards the pulmonary system. Although COVID-19 infection primarily manifests in the lungs, involvement of other organ systems, including the kidneys, has been highly documented. Such a mechanism results from organ crosstalk, causing the involvement of multiple organs and organ systems [1]. 

The impact of acute kidney injury (AKI) in COVID-19 in terms of patients’ mortality and morbidity is highly significant. Early reports from Chinese cohorts documented a low prevalence of renal involvement [2]. However subsequent reports, from the USA and Europe, indicate a much higher rate of AKI, particularly in the intensive-care setting, with up to 45% of patients in the intensive-care unit (ICU) requiring renal replacement therapy [3]. The reported incidence of AKI in COVID-19 has ranged from 0.5% to 27% among hospitalized patients [4,5]. Subsequent observational studies conducted in larger cohorts reported an incidence of AKI ranging from 0.5 to 10.4% [6]. Meanwhile, in a study by Chan et al. (2020), among 3993 patients with COVID-19, 39%, 19%, and 42% developed AKI stages 1, 2, and 3, respectively [7].

Mortality among hospitalized patients with COVID-19-associated AKI is higher than for those without kidney involvement [8]. Factors repeatedly associated with a higher risk of AKI in patients with COVID-19 include age, a history of hypertension, and diabetes mellitus. Chronic kidney disease (CKD) is a well-established risk factor for AKI in hospitalized patients and was shown to be the most relevant risk factor for AKI requiring renal replacement therapy [9]. In a meta-analysis by Wu et al. (2021), involving 42,779 COVID-19 patients, CKD has emerged as a prevalent comorbidity and carries the highest risk of severe COVID-19 [10]. Further investigation of this high-risk group is vital to improve outcomes and halt progression to end-stage renal failure.

AKI is increasingly being recognized as a negative prognostic factor in patients with COVID-19 due to its high incidence (19.76%) and mortality rate (54.24%) [11]. The occurrence of AKI was independently associated with increased mortality at 30 days post-admission, with a notably higher risk with increasing severity [12]. Identifying factors predictive for AKI may guide the management of patients—avoiding nephrotoxic drugs, careful hydration with careful observance, and timely initiation of dialysis—leading to better clinical outcomes and a reduction in long-term complications.

Currently, there is limited published literature in this area. Data on epidemiology, the need for renal support, and outcomes among COVID-19 patients with AKI are essential. In a resource-limited country such as the Philippines, where dialysis facilities may not be present in all hospitals, the identification of risk factors would facilitate the transfer of patients to institutions that can provide multi-organ support.

## 2. Materials and Methods

This study was carried out at the National Kidney and Transplant Institute (NKTI), Quezon City, Metro Manila, Philippines. The researchers utilized a retrospective cohort design. Non-probability consecutive sampling was performed. All patients aged ≥19 years old admitted to NKTI with COVID-19 from 1 March 2020 to 1 March 2022 were included. Kidney transplant patients, patients on chronic hemodialysis, patients who died within the first 24 h of admission, and patients who had dialysis initiation within 24 h from admission were excluded.

This study was approved by the Research Ethics Committee (REC) of NKTI on 7 June 2022 (approval code NKTI-REC-2022-40). Medical records were reviewed, and clinical, laboratory, and treatment data were obtained from each patient. Data were collected retrospectively. Post-hospital discharge outcomes were reviewed using outpatient medical records. Data collection was carried out by the researchers from June 2022 until September 2022. Variables were collected and recorded in Excel forms.

Stata MP version 17 software was used for data processing and analysis. Continuous variables were presented as mean (standard deviation/SD) or median (interquartile range/IQR). Categorical variables were presented as frequencies and percentages. The Kruskal–Wallis test was used to determine significance in distribution among the three groups. Significant results were further analyzed using Dunn’s test. Comparison of continuous variables was performed using the Kruskal–Wallis test and the Mann–Whitney U test. Categorical variables were compared using the chi-square test and Fisher’s exact test. A Kaplan–Meier survival curve was created to determine the probability of survival during hospital stay wherein Time 0 was the date of hospital admission. The log-rank test was used to compare the survival probability of patients with and without AKI. To determine the factors associated with AKI and in-hospital mortality, two logistic regression models were created. Simple logistic regression analysis, which generated the crude odds ratio (cOR), was performed to screen variables to be entered into the full regression model. Model building was performed using the backward elimination technique, and the adjusted ORs of significant variables were presented. *p* values ≤ 0.05 were considered statistically significant.

### Definition of Terms

1. Chronic kidney disease (CKD) was defined as abnormalities of kidney structure or function, present for more than 3 months. Criteria included at least one marker of kidney damage (Albuminuria (AER 30 mg/24 h; ACR > 30 mg/g), urine sediment abnormalities, electrolyte and other abnormalities due to tubular disorders, abnormalities detected by histology, structural abnormalities detected by imaging, history of kidney transplantation) or decreased eGFR (estimated glomerular filtration rate)— less than 60 mL/min/1.73 m^2^ (GFR categories G3a–G5) *(KDIGO 2012 Clinical Practice Guideline for the Evaluation and Management of Chronic Kidney Disease).*
2. COVID-19 confirmed case was defined as any individual, irrespective of presence or absence of clinical signs and symptoms, who was laboratory-confirmed for COVID-19 in a test conducted at the national reference laboratory, a subnational reference laboratory, and/or a Department of Health (DOH)-licensed COVID-19 testing laboratory. The laboratory test had to reveal a positive (a) real-time reverse transcriptase polymerase chain reaction (PCR) test or (b) geneXpert of oropharyngeal, nasopharyngeal, or endotracheal swab specimens.
3. Acute kidney injury (AKI) was defined according to the 2012 Kidney Disease: Improving Global Outcomes (KDIGO) criteria: (a) increase in serum creatinine (sCr) by ≥0.3 mg/dL (≥26.5 µmol/L) within 48 h; (b) increase in sCr to ≥1.5 times baseline, which is known or presumed to have occurred within the prior 7 days; or (c) urine volume < 0.5 mL/kg/h in 6 h.
  AKI stages were classified according to the 2012 Kidney Disease: Improving Global Outcomes (KDIGO) classification as follows: (a) Stage 1: increase in sCr ≥ 0.3 mg/dL within 48 h or 1.5 to 1.9 times increase in baseline sCr measured within 7 days; (b) Stage 2: 2–2.9 times increase of baseline sCr measured within 7 days; (c) Stage 3: 3 times or greater increase in baseline sCr measured within 7 days or at the point of initiation of renal replacement therapy (RRT). Baseline sCr was the last available sCr measurement within 365 days before the onset of COVID-19 symptoms. When not available prior to the diagnosis of COVID-19, sCr measurement on admission was used as the baseline value. AKI stage classification was the highest reached during hospitalization.
4. Charlson comorbidity index (CCI) was defined as a disease index to predict 10-year survival in patients with multiple comorbidities. It was the cumulative score based on presence of different comorbidities, scored as follows: 1 point for each of myocardial infarction, congestive heart failure, peripheral vascular disease, dementia, cerebrovascular disease, chronic lung disease, connective tissue disease, ulcer, chronic liver disease, diabetes; 2 points for each of hemiplegia, moderate or severe kidney disease, diabetes with end-organ damage, tumor, leukemia, lymphoma; 3 points for moderate or severe liver disease; and 6 points for tumor metastasis or AIDS [13].
5. Body mass index was calculated as weight in kilograms divided by the square of the height in meters (kg/m^2^) and was categorized into four groups according to the Asian-Pacific cutoff points: underweight (<18.5 kg/m^2^), normal weight (18.5–22.9 kg/m^2^), overweight (23–24.9 kg/m^2^), and obese (≥25 kg/m^2^).
6. COVID-19 severity was classified according to the latest DOH Advisory on COVID-19 Protocols for Quarantine and Isolation (January 2022): (a) Mild, showing no pneumonia or desaturation; (b) Moderate, with pneumonia but with no difficulty in breathing or shortness of breath, RR < 30 breaths/min, oxygen saturation > 94% at room air, or without pneumonia but with risk factors for progression: elderly (60 years old and above) and/or with comorbidities; (c) Severe, with pneumonia and any one of the following: signs of respiratory distress, oxygen saturation < 94% at room air, respiratory rate of >30 breaths/minute, requiring oxygen supplementation; (d) Critical, with pneumonia and any of the following: impending respiratory failure requiring high-flow oxygen, non-invasive or invasive ventilation, acute respiratory distress syndrome, sepsis or shock, deteriorating sensorium, multi-organ failure, and thrombosis. Pneumonia was defined as evidence of lower respiratory disease during clinical assessment (e.g., cough, fever plus crackles) and/or imaging (CXR, ultrasound, CT scan).
7. Renal recovery is defined by the Acute Disease Quality Initiative (ADQI) 16 consensus group as the absence of AKI according to both serum creatinine and urine output criteria (per KDIGO) within 7 days of AKI onset. AKI that has not resolved within a week is termed acute kidney disease. For this study, recovery from AKI was defined as the absence of any stage of AKI in the last-recorded creatinine during hospitalization (i.e., serum creatinine < 1.5 times the baseline creatinine), and in the absence of RRT. Follow-up creatinine at 7–14 days after discharge was retrieved, and the operational definition of renal recovery was utilized.
8. Renal replacement therapy (RRT) was defined as hemodialysis or peritoneal dialysis modality.

## 3. Results

- Baseline Characteristics

Included in the analysis were 519 patients diagnosed with COVID-19 during admission. The patients were classified as having diagnosed CKD (as stated on medical records, or where the criteria stated above in the definition of terms were met), undiagnosed CKD but with decreased eGFR at baseline (<90 mL/min/1.73 m^2^), or normal eGFR at baseline (≥90 mL/min/1.73 m^2^). Table 1 shows the patients’ baseline characteristics as compared between groups. Of these, 41% were diagnosed with CKD, 31% were undiagnosed with CKD but with reduced baseline eGFR, and 28% showed normal baseline eGFR. The male to female ratio in the diagnosed CKD group was roughly 3:2. The median age was 57 years old (range 19–92 years old). Patients with diagnosed CKD were significantly younger compared to those with undiagnosed CKD (*p* = 0.0013). The median Charlson comorbidity index (CCI) was 3 (range: 0–12). Further analysis showed that patients with normal eGFR at baseline had significantly lower comorbidities than patients with diagnosed CKD (*p* < 0.00001) and those with undiagnosed CKD (*p* < 0.00001). No significant differences between groups were noted in terms of smoking history, body mass index (BMI), baseline mean arterial pressure, and severity of acute respiratory distress syndrome (ARDS). A higher number of patients with diagnosed CKD needed inotropes. Patients in the diagnosed CKD group had notably higher levels of baseline ferritin, high-sensitivity C reactive protein (hsCRP), D dimer, and procalcitonin, and a higher white blood cell (WBC) count. Meanwhile, patients in the normal eGFR group had higher levels of baseline hemoglobin, albumin, and alanine transaminase (ALT), and a higher lymphocyte count. Critical COVID-19 developed in 26%, 24%, and 14% of the groups, respectively.

B. Incidence

The overall incidence of AKI was 45.66% (95% CI: 41.41–49.98%), with 77%, 39%, and 8% AKI incidence among diagnosed CKD, undiagnosed CKD, and normal eGFR, respectively. AKI incidences significantly differed among the three groups (*p <* 0.001). The percentages for patients who developed AKI stages 1, 2, and 3 were 35%, 9%, and 56%, respectively.

C. Risk Factors for AKI

For the regression analysis, the following variables were excluded from the analysis since most patients had missing data for these: LDH, ferritin, hsCRP, D dimer, procalcitonin, albumin, ALT, AST, and proteinuria. One patient with missing data on hemoglobin, WBC, and lymphocytes was excluded. The total number of patients included in the analysis was 518.

Male sex, CCI, the need for inotropes, ARDS, baseline WBC count, and COVID-19 severity were associated with increased odds of AKI among COVID-19 patients (Supplementary Table S1). Males had 52% higher odds of AKI compared to females (cOR 1.52, *p* < 0.019). For every 1-unit increase in CCI, the odds of AKI increased by 21% (cOR 1.21, *p* < 0.001). Patients who needed inotropes had four times higher odds of AKI than those who did not (cOR 4.04, *p* < 0.001). Patients with ARDS had three times higher odds of AKI (cOR 2.59, *p* < 0.004). For every 1 × 10^9^/L increase in WBC, the odds of AKI increased by about 4% (cOR 1.04, *p* = 0.003). Compared to mild cases, patients with critical COVID-19 had three times higher odds of AKI (cOR 3.32, *p* < 0.001).

BMI, hemoglobin, lymphocyte count, and baseline eGFR were found to be protective against AKI. Paradoxically, obese patients had 89% lower odds of AKI (cOR 0.53, *p* < 0.001). For every 1 g/dl increase in hemoglobin, the odds of AKI decreased by 20% (cOR 0.83, *p* < 0.0001). For every 1% increase in lymphocytes, the odds of AKI decreased by 5% (cOR 0.95, *p* < 0.001). For every 1 mL/min increase in eGFR, the odds of AKI decreased by about 4% (cOR 0.96, *p* < 0.001).

The variables that remained statistically significant (Table 2) in the multivariable analysis were male sex, need for inotropes, and baseline eGFR. Males had 78% higher odds of AKI compared to females (aOR 1.78, *p* < 0.018). Patients who used inotropes had three times higher odds of AKI than those who did not (aOR 2.84, *p* < 0.001). For every 1 ml/min increase in eGFR, the odds of AKI decreased by about 4% (aOR 0.96, *p* < 0.001).

As for the variables not included in the multivariate analysis, the researchers performed univariable analysis to assess association with AKI (Supplementary Table S2). For every 100 U/L increase in LDH, the odds of AKI increased by 9% (cOR 1.09, *p* = 0.017). For every 100 ng/mL increase in ferritin, the odds of AKI increased by 2% (cOR 1.02, *p* < 0.001). For every 100 mg/L increase in hsCRP, the odds of AKI increased by 77% (cOR 1.77, *p* < 0.001). For every 1 ug/mL increase in D dimer, the odds of AKI increased by 15% (cOR 1.15, *p* < 0.001). For every 1 ng/mL increase in procalcitonin, the odds of AKI increased by 16% (cOR 1.16, *p* < 0.001). In contrast, for every 1 g/dL increase in albumin, the odds of AKI decreased twofold (cOR 0.46, *p* < 0.001).

D. In-Hospital Outcomes

Table 3 shows that 48.5% of patients with AKI needed renal replacement therapy (RRT). AKI was associated with a higher need for mechanical ventilation compared to those without AKI (*p* < 0.001). A total of 111 (21%) patients died during the study period. The mortality rate among AKI patients was significantly higher than among those without AKI (*p* < 0.001). There was no difference in mortality, however, among patients with CKD, those with undiagnosed CKD with abnormal baseline eGFR, and those with normal baseline eGFR (*p* = 0.802). Among the 408 patients who survived until discharge, the median length of stay (LOS) was 10 days with a range of 0–121 days and was significantly longer in patients with AKI than in those without AKI (*p* = 0.001).

**Figure 1:**
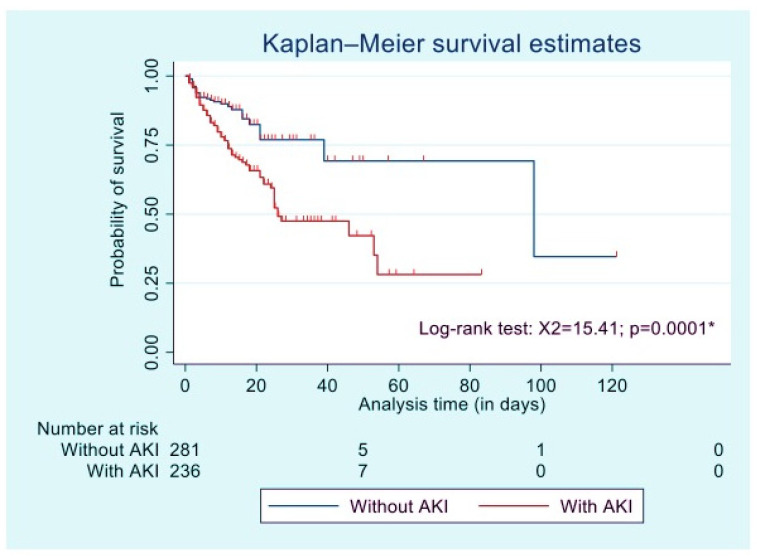
Kaplan–Meier survival curve: patient in-hospital survival among patients with and without AKI. * *p* value < 0.05.

The KM curve (Figure 1) shows that the probability of survival at any time point significantly differed between patients with and without AKI (*p* = 0.001). Survivor probability at 7, 14, and 30 days was 91.27% without AKI vs. 83.16% with AKI; 87.82% without AKI vs. 70.59% with AKI; and 76.95% without AKI vs. 47.50% with AKI, respectively. The median time to death was 7 days [IQR: 3–15; Range: 1–98].

E. Renal Recovery

Renal recovery among COVID-19 patients with AKI is presented in Table 4. Overall renal recovery was 41%: 64%, 59%, and 23% for AKI Stages 1, 2, and 3, respectively. Stage 3 AKI had a significantly lower recovery rate compared with other stages (*p* < 0.001).

Among the 115 patients who underwent RRT (Table 5) during their hospital stay, only 20% recovered. Renal recovery was significantly higher in patients who did not undergo RRT (59% vs. 20%, *p* < 0.001).

F. Outcomes Post-Discharge

Of the 408 patients who survived until discharge, only 232 patients had data on 7–14 days post-discharge. No additional deaths were recorded. Of the 159 AKI patients who survived until discharge, only 115 had data on renal recovery post-discharge; 43% of these patients had renal recovery but showed variation in AKI severity: 62%, 100%, and 26% for AKI stages 1, 2, and 3, respectively. A significant difference was observed between renal recovery and AKI staging post-discharge (*p* < 0.001). Renal recovery post-discharge was significantly lower among patients who underwent RRT (20% vs. 62%, *p* < 0.001).

G. Risk Factors for In-Hospital Mortality

Univariable analysis (Supplementary Table S3) showed that age, being male, inotrope use, ARDS, and COVID-19 severity were significantly associated with in-hospital mortality among COVID-19 patients with AKI. For every 1-year increase in age, the odds of in-hospital mortality increased by 2% (cOR 1.02, *p* = 0.022). Males had 85% lower odds of in-hospital mortality than females (cOR 0.54, *p* = 0.029). Patients who needed inotropes had about eight times higher odds of mortality than those who did not (cOR 8.47, *p* < 0.001). Mild, moderate, and severe ARDS had higher odds of mortality. Severe, moderate, and critical cases had 5, 11, and 73 times higher odds of mortality, respectively (cOR 5.03, *p* = 0.047; cOR 10.81, *p* = 0.003; cOR 72.55, *p* < 0.001). In contrast, for every 1% increase in lymphocytes, the odds of in-hospital mortality decreased by 6% (cOR 0.94, *p* = 0.003).

The variables that remained statistically significant (Table 6) in multivariable analysis were inotrope use and lymphocyte count. Patients who needed inotropes had about nine times higher odds of mortality than those who did not (aOR 8.59, *p* < 0.001), while for every 1% increase in lymphocyte count, the odds of in-hospital mortality decreased by 6% (aOR 0.94, *p* < 0.003).

Univariable analysis of the excluded variables (Supplementary Table S4) showed that LDH, hsCRP, albumin, and AST were significantly associated with in-hospital mortality among patients with AKI. For every 100 U/L increase in LDH, the odds of in-hospital mortality increased by 21% (cOR 1.21, *p* < 0.001). For every 100 mL/L increase in hsCRP, the odds of in-hospital mortality increased by 83% (cOR 1.83, *p* = 0.001). For every 1 g/dL increase in albumin, the odds of in-hospital mortality decreased by 69% (cOR 0.59, *p* = 0.011). For every 1 IU/L increase in AST, the odds of in-hospital mortality increased by 1% (cOR 1.01, *p* = 0.001).

## 4. Discussion

Several studies [14,15] have established the damaging effects of COVID-19 on the kidneys, leading to a progressive decline in kidney function and eventual chronic kidney disease.

In this study, the overall incidence of AKI in COVID-19 was 45.66%. This was very high compared to the systematic review by Raina et al. (2022) involving 60 studies with a population of 42,612 and showing a pooled AKI incidence of 19.45% [15]. The higher AKI incidence in our institution may be due to NKTI being a tertiary renal referral center and 41% of the cohort in this study being known CKD not on dialysis compared to only 5.2% in the meta-analysis. CKD is an established risk factor for AKI development, thus explaining the higher incidence rate.

The CKD cohort in this study showed a higher CCI, higher inotrope use and levels of baseline ferritin, hsCRP, D dimer, and procalcitonin, and higher WBC counts compared to the undiagnosed CKD and normal eGFR cohorts. This predominantly renal population had more severe baseline characteristics compared to the cohorts of Raina et al. [15]. This study emphasized the high incidence of AKI in a CKD population (77%), compared to patients with undiagnosed CKD but with low baseline eGFR (39%) and those with normal eGFR (8%). The meta-analyses of Cai et al. (2021) [10] and Daniella et al. (2021) [11] both showed that underlying CKD was strongly associated with AKI. Damage to viable nephrons leads to a reduction in renal functional reserve, predisposing to AKI.

In this study, patients developed AKI stages 1, 2, and 3 in the proportions 35%, 9%, and 56%, respectively, which was comparable to a large-scale retrospective cohort study by Chan et al. (2020), involving 3993 hospitalized patients with COVID-19 and showing 39%, 19%, and 42%, respectively [7,16].

The pathophysiology of COVID-19 AKI involves very robust local and systemic inflammatory and immune responses leading to endothelial injury and activation of coagulation and the renin–angiotensin system evidenced by a rise in inflammatory markers [17]. Univariate analysis of the inflammatory markers in this study showed that LDH (*p* = 0.017), ferritin (*p* < 0.0001), hsCRP (*p* < 0.001), D dimer (*p* < 0.001), and procalcitonin (*p* < 0.001) significantly increased the odds of AKI while albumin decreased it (*p* < 0.001). This was comparable to a study by Ortiz et al. (2021) involving 644 patients with COVID-19 and showing significantly higher ferritin (*p* < 0.001), CRP (*p* < 0.001), procalcitonin (*p* < 0.001), D dimer (*p* = 0.004) and LDH (*p* = 0.004) among patients with AKI compared to those without AKI. In the same study, multivariate analysis showed that CRP (*p* = 0.003) and ferritin (*p* < 0.035) were significantly associated with AKI [18]. In addition, Naser et al. (2021) showed that hypoalbuminemia predicted the outcome of COVID-19, independent of age and comorbidity. Mortality was correlated with the severity of hypoalbuminemia [19].

In this study, multivariate analysis of other risk factors for AKI showed that being male (*p* = 0.018) and the need for inotropes (*p* < 0.001) were significantly associated with AKI. On the other hand, an increase in eGFR (*p* < 0.001) was found to be protective. CCI, baseline WBC, ARDS, and COVID-19 severity were not found to be significant. This was comparable to the studies by Hirsch et al. (2020) and He et al. (2022) in which being male was likewise an independent predictor for AKI in COVID-19 [20,21]. Androgens (AR) found in males regulate the expression of angiotensin-converting enzyme-2 (ACE-2) and transmembrane serine protease 2 (TMPRSS-2), predisposing severe COVID-19 infection. AR increase efferent arteriolar resistance by increasing the level of angiotensin II, resulting in glomerular injury and progression of kidney damage [21]. Alfano et al. (2021) showed that the use of inotropes was associated with AKI in COVID-19 patients since they lead to intrarenal vasoconstriction, decreased renal perfusion, and eGFR [22].

This study found that AKI in COVID-19 was associated with a higher need for invasive ventilation (*p* < 0.001), an increased length of stay (*p* < 0.001), and the need for dialysis. The systematic review of Raina et al. (2022) [15] showed that the proportion of AKI in COVID-19 patients needing dialysis was 39.04% in comparison to 48.5% as found in this study. The higher requirement for dialysis in this study may be due to the higher CKD population of 41% compared to 5.2% and the higher AKI incidence of 45.6% compared to 19.45% in the meta-analysis.

Overall renal recovery was 41% in this study and was less likely with increasing AKI stage (*p* < 0.001). Renal recovery was likewise significantly higher in patients who did not undergo RRT (*p* < 0.001). Renal recovery was low compared to the study of Jewel et al. (2021) [12] involving 1248 patients and with a recovery rate of 84%. The lower recovery rate in this study was expected, since 56% of the AKI cohort had stage 3 AKI compared to only 36% in the Jewel et al. cohort. Moreover, 48.5% needed dialysis in this study compared to only 22% in the Jewel et al. cohort.

In this study, the mortality rate was significantly higher in patients with AKI compared to patients without AKI (*p* < 0.001). This was comparable to the systematic review by Raina et al. (2022) [15]. There was no difference in mortality, however, among patients with CKD, those with undiagnosed CKD with abnormal baseline eGFR, and those with normal baseline eGFR (*p* = 0.802). This was comparable to the study of Naser et al. (2021) [19], which showed that death was not associated with a history of CKD (*p* > 0.5). This was unexpected, since CKD is a known risk factor for mortality. This may be due to a very short observation period of 9 days for this study.

Multivariate analysis in this study showed that the need for inotropes increased the odds for in-hospital mortality (*p* < 0.001), while an increase in lymphocyte count was protective (*p* = 0.0001). Age, being male, ARDS, and COVID-19 severity were not risk factors. Looking at the inflammatory markers, univariable analysis found that LDH (*p* < 0.001), hsCRP (*p* = 0.001), and AST(*p* = 0.001) increased the odds of in-hospital mortality, while albumin decreased it (*p* = 0.011). These results were comparable to the meta-analyses of Cai et al. (2021) [10] and Daniella et al. (2021) [11].

## 5. Conclusions

The incidence of AKI was 78% in a population with established CKD compared with 38% in those with undiagnosed CKD but with abnormal eGFR at baseline and 7% in those with normal eGFR. Male sex and the need for inotropes were risk factors for AKI, while increased baseline eGFR was protective. AKI leads to poor patient survival, a higher need for invasive ventilation, an increased length of hospital stay, and a decreased chance of renal recovery, especially among those with CKD. The need for inotropes was a risk factor for in-hospital mortality, while an increase in lymphocyte count was protective. Prevention, early recognition and management of AKI, especially in a high-risk CKD population, is essential to improved renal recovery and patient survival.

## 6. Recommendations

Further studies involving longer patient follow-up should be performed to determine the risk of further deterioration of renal function and progression to end-stage renal disease. Inflammatory markers should be obtained in all patients hospitalized for COVID-19 to establish early markers for AKI development.

## 7. Limitations

These findings need to be validated in a large multicenter cohort. This was a retrospective study; thus, specific inferences regarding causality could not be made. The data on inflammatory markers were incomplete as they are not routinely requested; thus, multivariable analysis was not pursued.

## Figures and Tables

**Table 1:** Demographic Characteristics of COVID-19 patients (*n* = 519).

	All (*n* = 519)	Diagnosed CKD (*n* = 214)	Undiagnosed CKD but Decreased eGFR on Baseline (*n* = 160)	Normal eGFR (*n* = 145)	*p*
	Mean ± SD; Median (IQR); Frequency (%)				
Age (years), median	57 [IQR: 45–68]	58 [IQR: 47–68]	65 [IQR: 50–75]	48 [IQR: 35–58]	<0.001 ^a^
Sex					
Male	289 (56)	130 (61)	92 (58)	67 (46)	0.021 ^b^
Female	230 (44)	84 (39)	68 (42)	78 (54)	
Charlson Comorbidity Index, median	3 [IQR: 1–5]	4 [IQR: 2–6]	3 [IQR: 1–6]	1 [IQR: 0–3]	<0.001 ^a^
Smoking History					
No	439 (85)	186 (87)	127 (79)	126 (87)	0.279 ^b^
Yes, current smoker	31 (6)	12 (6)	12 (8)	7 (5)	
Yes, former smoker	49 (9)	16 (7)	21 (13)	12 (8)	
BMI (kg/m^2^), median	23.85 [IQR: 21.08–27.34]	23.60 [IQR: 20.71–26.82]	23.53 [IQR: 21.02–27.45]	24.38 [IQR: 21.64–27.34]	0.173 ^a^
Underweight	46 (9)	19 (9)	18 (11)	9 (6)	0.161 ^b^
Normal	168 (32)	73 (34)	57 (36)	38 (26)	
Overweight	104 (20)	45 (21)	24 (15)	35 (24)	
Obese	201 (39)	77 (36)	61 (38)	63 (44)	
Baseline Clinical Status					
MAP (mmHg), median	91.33 [IQR: 78.33–102]	93.33 [IQR: 75.67–107.67]	90 [IQR: 69.33–100]	90.33 [IQR:80–100.33]	0.761 ^a^
On inotropes, %yes	184 (35)	98 (46)	59 (37)	27 (19)	<0.001 ^b^
PaO2/Fio2 ratio	404.76 [IQR: 230–476.19]	423.81 [IQR: 257.14–476.19]	376.19 [IQR: 191.31–476.19]	385.71 [IQR: 298–476.19]	0.022 ^a^
Normal	358 (69)	156 (73)	96 (60)	106 (73)	0.058 ^b^
Mild	48 (9)	14 (7)	19 (12)	15 (10)	
Moderate	67 (13)	29 (14)	26 (16)	12 (8)	
Severe	46 (9)	15 (7)	19 (12)	12 (8)	
Baseline Inflammatory Markers					
LDH, median (*n* = 403)	333 [IQR: 234–506]	342 [IQR: 227–534]	329 [IQR: 236.50–490.50]	332.50 [IQR: 235.50–513.50]	0.826 ^a^
Serum ferritin, median (*n* = 408)	1170.42 [IQR: 543.02–2925.24]	1374 [IQR: 617.89–3302.60]	1265.48 [IQR: 657.67–2975.04]	779.01 [IQR: 352.07–2452.16]	0.003 ^a^
HSCRP, median (*n* = 398)	73.86 [IQR: 33.81–128.72]	91.98 [IQR: 42.56–138.72]	72.77 [IQR: 42.16–135.79]	50.18 [IQR: 14.40–104.38]	<0.001 ^a^
D dimer, median (*n* = 363)	1.68 [IQR: 0.83–3.78]	2.89 [IQR: 1.40–4.78]	1.77 [IQR: 0.96–3.79]	0.89 [IQR: 0.50–1.50]	<0.001 ^a^
Procalcitonin, median (*n* = 410)	0.27 [IQR: 0.09–1.34]	1.18 [IQR: 0.29–4.59]	0.19 [IQR: 0.09–0.63]	0.11 [IQR: 0.05–0.27]	<0.001 ^a^
Baseline Laboratories					
Hemoglobin(g/dL), median (*n* = 518)	12.15 [IQR: 9.70–14.10]	10.80 [IQR: 8.80–13]	12.50 [IQR: 10.20–14.15]	13.60 [IQR: 12.10–14.90]	<0.001 ^a^
WBC(×10^9^/L), median (*n* = 518)	9.13 [IQR: 6.23–13.84]	10.31 [IQR: 7.26–15.55]	9.49 [IQR: 6.25–13.57]	7.60 [IQR: 5.33–11.64]	<0.001 ^a^
Lymphocyte(%), median (*n* = 518)	0.12 [IQR: 0.08–0.21]	0.10 [IQR: 0.06–0.17]	0.12 [IQR: 0.09–0.20]	0.17 [IQR: 0.10–0.24]	<0.001 ^a^
eGFR(umol/L) measured	56.91 [IQR: 16.67–93.82]	14.31 [IQR: 6.91–32.53]	66.20 [IQR: 46.95–77.25]	105.49 [IQR: 97.44–116.50]	<0.001 ^a^
S. Albumin(g/dL) (*n* = 474)	3.50 [IQR: 2.97–4.00]	3.30 [IQR: 2.80–3.71]	3.51 [IQR: 3.06–3.97]	3.87 [IQR: 3.50–4.31]	<0.001 ^a^
AST(IU/L) (*n* = 414)	50.50 [IQR: 31–83]	45 [IQR: 28–80]	54 [IQR: 32–84]	55.50 [IQR: 32–85]	0.201 ^a^
ALT(IU/L) (*n* = 440)	37 [IQR: 20–72.50]	32 [IQR: 17–55]	37 [IQR: 21–77]	50 [IQR: 26–80]	<0.001 ^a^
Baseline proteinuria (*n* = 388)	378 (97)	169 (99)	114 (93)	95 (99)	0.006 ^c^
COVID-19 Severity					
Mild	148 (29)	66 (31)	40 (25)	42 (29)	0.001 ^b^
Moderate	134 (26)	62 (29)	34 (21)	38 (26)	
Severe	124 (24)	31 (14)	48 (30)	45 (31)	
Critical	113 (22)	55 (26)	38 (24)	20 (14)	

^a^ Kruskal–Wallis test was used; significant results were further analyzed using Dunn’s test. ^b^ Chi-square test was used. ^c^ Fisher’s exact test was used.

**Table 2:** Factors associated with acute kidney injury among COVID-19 patients (*n* = 518).

	Adjusted OR (95% CI)	*p* < 0.05
Age (years)	-	-
Sex		
Female	*Ref*	*Ref*
Male	1.78 (1.10–2.87)	0.018
Baseline Clinical Status		
On inotropes	2.84 (1.75–4.60)	<0.001
Baseline Laboratories		
Lymphocyte	-	-
eGFR (measured)	0.96 (0.95–0.96)	<0.001

**Table 3:** Association between AKI and need for RRT, invasive ventilation, and prolonged stay.

	All (*n* = 519)	Acute Kidney Injury		*p*
		With (*n* = 237)	Without (*n* = 282)	
	Mean ± SD; Median (IQR); Frequency (%)			
Need for RRT, %yes	115 (22)	115 (48.5)	0 (0)	
Need for mechanical ventilation	103 (20)	75 (32)	28 (10)	<0.001 ^a^
Length of hospital stay (days) (*n* = 408)	10 [IQR: 6–16]	8 [IQR: 4–16]	4 [IQR: 2–12]	0.001 ^b^
Mortality	111 (21)	78 (33)	33 (12)	<0.001 ^b^

^a^ Chi-square test was used; ^b^ Mann–Whitney U test was used.

**Table 4:** Proportion of AKI patients who had in-hospital renal recovery among COVID-19 patients (*n* = 237).

	N	Renal Recovery (Based on ADQI) *n* (%)	Renal Recovery (Based on Operational Definition) *n* (%)	Not Recovered *n* (%)
All AKI patients	237	17 (7.17)	79 (33.33)	141 (59)
Stage 1	83	8 (10)	45 (54)	30 (36)
Stage 2	22	3 (14)	10 (45)	9 (41)
Stage 3	132	6 (5)	24 (18)	102 (77)

(Chi-square test: *p* value < 0.001.)

**Table 5:** In-hospital renal recovery comparison among patients with and without RRT.

RRT	Recovered (N = 95) *n* (%)	Did Not Recover (N = 142) *n* (%)
Yes	23 (20)	92 (80)
No	72 (59)	50 (41)

(Chi-square test: *p* < 0.001.)

**Table 6:** Factors associated with in-hospital mortality among COVID-19 patients with acute kidney injury (*n* = 237).

	Adjusted OR (95% CI)	*p* Value ≤0.05
Baseline Clinical Status		
On inotropes (Ref: No)	8.59 (4.30–17.16)	<0.001
Baseline Laboratories		
Lymphocyte	0.94 (0.90–0.98)	0.003

## Data Availability

No new data were created or analyzed in this study. Data sharing is not applicable in this article.

## Supplementary Materials

The following supporting information can be downloaded at: https://www.mdpi.com/article/10.3390/tropicalmed8080387/s1, Table S1: Factors associated with acute kidney injury among COVID-19 patients; Table S2: Association of selected factors with acute kidney injury among COVID-19 patients; Table S3: Factors associated with in-hospital mortality among COVID-19 patients with AKI; Table S4: Association of selected factors with in-hospital mortality among COVID-19 patients with AKI.

## References

[1] Ronco C., Reis T. (2020). Kidney involvement in COVID-19 and rationale for extracorporeal therapies. Nat. Rev. Nephrol..

[2] Huang C., Wang Y., Li X., Ren L., Zhao J., Hu Y., Zhang L., Fan G., Xu J., Gu X. (2020). Clinical features of patients infected with 2019 novel coronavirus in Wuhan, China. Lancet.

[3] Ng J.H., Hirsch J.S., Hazzan A., Wanchoo R., Shah H.H., Malieckal D.A., Ross D.W., Sharma P., Sakhiya V., Fishbane S. (2021). Outcomes among Patients Hospitalized with COVID-19 and Acute Kidney Injury. Am. J. Kidney Dis..

[4] Cheng Y., Luo R., Wang K., Zhang M., Wang Z., Dong L., Li J., Yao Y., Ge S., Xu G. (2020). Kidney disease is associated with in-hospital death of patients with COVID-19. Kidney Int..

[5] Alenezi F.K., Almeshari M.A., Mahida R., Bangash M.N., Thickett D.R., Patel J.M. (2021). Incidence and risk factors of acute kidney injury in COVID-19 patients with and without acute respiratory distress syndrome (ARDS) during the first wave of COVID-19: A systematic review and Meta-Analysis. Ren. Fail..

[6] Pei G., Zhang Z., Peng J., Liu L., Zhang C., Yu C., Ma Z., Huang Y., Liu W., Yao Y. (2020). Renal Involvement and Early Prognosis in Patients with COVID-19 Pneumonia. J. Am. Soc. Nephrol..

[7] Chan L., Chaudhary K., Saha A., Chauhan K., Vaid A., Zhao S., Paranjpe I., Somani S., Richter F., Miotto R. (2021). AKI in Hospitalized Patients with COVID-19. J. Am. Soc. Nephrol..

[8] Gupta S., Coca S.G., Chan L., Melamed M.L., Brenner S.K., Hayek S.S., Sutherland A., Puri S., Srivastava A., Leonberg-Yoo A. (2021). AKI Treated with Renal Replacement Therapy in Critically Ill Patients with COVID-19. J. Am. Soc. Nephrol..

[9] Nimkar A., Naaraayan A., Hasan A., Pant S., Durdevic M., Suarez C.N., Elenius H., Hambardzumyan A., Lakshmi K., Mandel M. (2020). Incidence and Risk Factors for Acute Kidney Injury and Its Effect on Mortality in Patients Hospitalized From COVID-19. Mayo Clin. Proc. Innov. Qual. Outcomes.

[10] Cai X., Wu G., Zhang J., Yang L. (2021). Risk Factors for Acute Kidney Injury in Adult Patients With COVID-19: A Systematic Review and Meta-Analysis. Front. Med..

[11] Daniella D., Kandarini Y., Mahadita G.W. (2021). Risk Factors for Acute Kidney Injury in COVID-19 Patients: A Systematic Review. Open Access Maced. J. Med. Sci..

[12] Jewell P.D., Bramham K., Galloway J., Post F., Norton S., Teo J., Fisher R., Saha R., Hutchings S., Hopkins P. (2021). COVID-19-related acute kidney injury; incidence, risk factors and outcomes in a large UK cohort. BMC Nephrol..

[13] Charlson M.E., Pompei P., Ales K.L., MacKenzie C.R. (1987). A new method of classifying prognostic comorbidity in longitudinal studies: Development and validation. J. Chronic Dis..

[14] Fisher M., Neugarten J., Bellin E., Yunes M., Stahl L., Johns T.S., Abramowitz M.K., Levy R., Kumar N., Mokrzycki M.H. (2020). AKI in Hospitalized Patients with and without COVID-19: A Comparison Study. J. Am. Soc. Nephrol..

[15] Raina R., Mahajan Z.A., Vasistha P., Chakraborty R., Mukunda K., Tibrewal A., Neyra J.A. (2022). Incidence and Outcomes of Acute Kidney Injury in COVID-19: A Systematic Review. Blood Purif..

[16] Golmai P., Larsen C.P., DeVita M.V., Wahl S.J., Weins A., Rennke H.G., Bijol V., Rosenstock J.L. (2020). Histopathologic and Ultrastructural Findings in Postmortem Kidney Biopsy Material in 12 Patients with AKI and COVID-19. J. Am. Soc. Nephrol..

[17] Legrand M., Bell S., Forni L., Joannidis M., Koyner J.L., Liu K., Cantaluppi V. (2021). Pathophysiology of COVID-19-associated acute kidney injury. Nat. Rev. Nephrol..

[18] Ortiz A.E.T., Walker J.B., Mohammed A.E., Mohamed M., Lukitsch I., Velez J.C.Q. (2020). Markers of inflammation and risk for AKI and need for dialysis in patients with COVID-19. J. Am. Soc. Nephrol..

[19] Naser M.N., Al-Ghatam R., Darwish A.H., Alqahtani M.M., Alahmadi H.A., Mohamed K.A., Hasan N.K., Perez N.S. (2021). Risk factors, predictions, and progression of acute kidney injury in hospitalized COVID-19 patients: An observational retrospective cohort study. PLoS ONE.

[20] Hirsch J.S., Ng J.H., Ross D.W., Sharma P., Shah H.H., Barnett R.L., Hazzan A.D., Fishbane S., Jhaveri K.D., Northwell COVID-19 Research Consortium, & Northwell Nephrology COVID-19 Research Consortium (2020). Acute kidney injury in patients hospitalized with COVID-19. Kidney Int..

[21] He W., Liu X., Hu B., Li D., Chen L., Li Y., Zhu K., Tu Y., Xiong S., Wang G. (2022). Gender and Ethnic Disparities of Acute Kidney Injury in COVID-18. Front. Cell. Infect. Microbiol..

[22] Alfano G., Ferrari A., Fontana F., Mori G., Magistroni R., Meschiari M., Franceschini E., Menozzi M., Cuomo G., Orlando G. (2021). Incidence, risk factors and outcome of acute kidney injury (AKI) in patients with COVID-19. Clin. Exp. Nephrol..

