# Supplementary material for: Incidence of, and Risk Factors and Outcomes Associated with, Acute Kidney Injury in COVID-19 at the National Kidney and Transplant Institute, Philippines
Source: Trop Med Infect Dis. 2023 Jul 28;8(8):387. doi: 10.3390/tropicalmed8080387 (PMC10459419; doi:10.3390/tropicalmed8080387)
Supplement: Supplementary file 1 [file tropicalmed-08-00387-s001.zip › tropicalmed-2466846-supplementary.pdf]

Supplemental Tables

Table S1. Factors associated with acute kidney injury among COVID-19 patients (n=518).

	**Crude OR (95% CI)**	**P value**	**Adjusted OR (95% CI)**	***p* value <0.05**
If Age (years)	1.01 (1.00-1.02)	0.132	*Removed*	*Removed*
Sex				
Female	*Ref*	*Ref*	***Ref***	***Ref***
Male	1.52 (1.07-2.16)	0.019	**1.78 (1.10-2.87)**	**0.018**
Charlson Comorbdity Index	1.21 (1.13-1.31)	<0.001	*Removed*	*Removed*
Smoking History				
No	*Ref*	*Ref*	*Removed*	*Removed*
Yes, current smoker	1.53 (0.73-3.24)	0.261	*Removed*	*Removed*
Yes, former smoker	0.68 (0.37-1.25)	0.218	*Removed*	*Removed*
BMI (Asian Pacific Classification)				
Normal	*Ref*	*Ref*	*Removed*	*Removed*
Underweight	0.88 (0.46-1.71)	0.715	*Removed*	*Removed*
Overweight	0.67 (0.41-1.10)	0.112	*Removed*	*Removed*
Obese	0.53 (0.35-0.80)	0.002	*Removed*	*Removed*
Baseline Clinical Status				
MAP	1.00 (0.99-1.00)	0.324	*Removed*	*Removed*
On Inotropes (Ref: No)	4.04 (2.76-5.93)	<0.001	**2.84 (1.75-4.60)**	**<0.001**
PaO2/Fio2 ratio: ARDS				
Normal	*Ref*	*Ref*	*Removed*	*Removed*
Mild	1.17 (0.64-2.14)	0.616	*Removed*	*Removed*
Moderate	1.51 (0.89-2.55)	0.123	*Removed*	*Removed*
Severe	2.59 (1.36-4.92)	0.004	*Removed*	*Removed*
Baseline Laboratories				
Hemoglobin	0.83 (0.78-0.88)	<0.001	*Removed*	*Removed*
WBC	1.04 (1.01-1.06)	0.003	*Removed*	*Removed*
Lymphocyte	0.95 (0.93-0.97)	<0.001	*Removed*	*Removed*
eGFR (measured)	0.96 (0.95-0.96)	<0.001	**0.96 (0.95-0.96)**	**<0.001**

COVID-19 severity				
Mild	*Ref*	*Ref*	*Removed*	*Removed*
Moderate	1.21 (0.75-1.94)	0.432	*Removed*	*Removed*
Severe	0.71 (0.43-1.17)	0.180	*Removed*	*Removed*
Critical	3.32 (1.98-5.58)	<0.001	*Removed*	*Removed*

Table S2. Association of selected factors with acute kidney injury among COVID-19 patients.

	**Crude OR (95% CI)**	**P value**
LDH (n=403)	1.09 (1.02-1.17)	0.017
Serum Ferritin (n=408)	1.02 (1.01-1.02)	<0.001
hsCRP (n=398)	1.77 (1.34-2.33)	<0.001
D Dimer (n=363)	1.15 (1.08-1.23)	<0.001
Procalcitonin (n=410)	1.16 (1.08-1.25)	<0.001
S. Albumin (n=474)	0.46 (0.35-0.60)	<0.001
AST (n=414)	1.00 (1.00-1.00)	0.349
ALT (n=440)	1.00 (1.00-1.00)	0.939
Baseline Proteinuria (n=388)		
No	*Ref*	*Ref*
Yes	1.42 (0.40-5.12)	0.590

Ref: Reference category.

Table S3. Factors associated with in-hospital mortality among COVID-19 patients with AKI (n=237).

	**Crude OR (95% CI)**	***p* value**	**Adjusted OR (95% CI)**	***p* value**
Age (years)	1.02 (1.00-1.04)	0.022	*Removed*	*Removed*
Sex				
Female	*Ref*	*Ref*	*Removed*	*Removed*
Male	0.54 (0.31-0.94)	0.029	*Removed*	*Removed*
Charlson Comorbdity Index	1.07 (0.96-1.19)	0.247	*Removed*	*Removed*
Smoking History				
No	*Ref*	*Ref*	*Removed*	*Removed*
Yes, current smoker	0.65 (0.20-2.07)	0.464	*Removed*	*Removed*
Yes, former smoker	2.11 (0.80-5.56)	0.132	*Removed*	*Removed*
BMI (Asian Pacific Classification)				
Normal	*Ref*	*Ref*	*Removed*	*Removed*
Underweight	1.73 (0.68-4.42)	0.251	*Removed*	*Removed*
Overweight	0.71 (0.31-1.59)	0.402	*Removed*	*Removed*
Obese	1.40 (0.72-2.58)	0.348	*Removed*	*Removed*
Baseline Clinical Status				
MAP	0.99 (0.97-1.00)	0.060	*Removed*	*Removed*
On Inotropes (Ref: No)	8.47 (4.31-16.68)	<0.001	8.59 (4.30-17.16)	<0.001
PaO2/Fio2 ratio: ARDS				
Normal	*Ref*	*Ref*	*Removed*	*Removed*
Mild	5.01 (1.97-12.71)	0.001	*Removed*	*Removed*
Moderate	7.06 (3.18-15.66)	<0.001	*Removed*	*Removed*
Severe	4.17 (1.83-9.50)	0.001	*Removed*	*Removed*
Baseline Laboratories				
Hemoglobin	1.00 (0.92-1.09)	0.989	*Removed*	*Removed*
WBC	1.00 (0.98-1.02)	0.921	*Removed*	*Removed*
Lymphocyte	0.94 (0.91-0.98)	0.003	0.94 (0.90-0.98)	0.003
eGFR (measured)	1.01 (1.00-1.02)	0.085	*Removed*	*Removed*
COVID-19 severity				
Mild	*Ref*	*Ref*	*Removed*	*Removed*
Moderate	5.03 (1.04-24.37)	0.045	*Removed*	*Removed*

Severe	10.81 (2.25-52.04)	0.003	*Removed*	*Removed*
Critical	72.55 (16.29-323.11)	<0.001	*Removed*	*Removed*

Ref: Reference category.

Table S4. Association of selected factors with in-hospital mortality among --19 patients with AKI.

	**Crude OR (95% CI)**	***p* value**
LDH (n=189)	1.21 (1.09-1.35)	<0.001
Serum Ferritin (n=188)	1.003 (1.00-1.00)	0.219
HSCRP (n=186)	1.83 (1.28-2.63)	0.001
D Dimer (n=165)	0.98 (0.91-1.05)	0.593
Procalcitonin (n=193)	1.00 (1.00-1.01)	0.972
S. Albumin (n=230)	0.59 (0.40-0.89)	0.011
AST (n=188)	1.01 (1.003-1.01)	0.001
ALT (n=196)	1.00 (1.00-1.00)	0.267
Baseline Proteinuria (n=188)		
No	*Ref*	*Ref*
Yes	0.40 (0.06-2.95)	0.372

Ref: Reference category
